# Sex, Drugs, and Seizures: Subarachnoid Hemorrhage in a Young Patient

**DOI:** 10.7759/cureus.5929

**Published:** 2019-10-17

**Authors:** Alex Davis, Eric Cortez, Andrew Kalnow

**Affiliations:** 1 Emergency Medicine, OhioHealth Doctors Hospital, Columbus, USA

**Keywords:** hemorrhagic stroke, seizure, illicit drug use, emergency medicine, neuro critical care, critical care

## Abstract

A 17-year-old male patient presented to the emergency department (ED) with seizures after sexual intercourse. The patient was found to have an intracerebral hemorrhage (ICH) likely secondary to sexual intercourse and concomitant amphetamine use, an extremely rare finding in this patient population. In this case review, we will discuss the presentation, management, and disposition of subarachnoid hemorrhage (SAH), a well-known emergency diagnosis within the ED, while highlighting a case that is clearly uncommon. In addition, we discuss the etiology of ICH in the setting of intercourse and amphetamine use with the ultimate goal of understanding the interdisciplinary care of a complex subject.

## Introduction

Intracranial hemorrhage (ICH), more specifically, subarachnoid hemorrhage (SAH), is a finding in the emergency department (ED) that requires proper identification and action. As ED physicians, we stabilize patients on a daily basis; however, understanding why a bleed has occurred and what can lead to neurological deterioration allows us to make decisions that are most beneficial for the patient [1]. Rarely will this disease process present as a young male presenting with seizures with a substance abuse history. Amphetamines lead to a nearly five times increased risk of hemorrhagic stroke [2]. Etiology of ICH secondary to amphetamine use is uncertain, given the paucity of case studies within the literature; however, vasculitis-inducing luminal irregularities and acute elevation in blood pressure have been postulated [3-4]. While both sexual intercourse and amphetamine use are well documented as causes for hemorrhagic stroke, there is a dearth of literature discussing this in a young adult. Herein, we have presented a rare case study of a young male patient who suffered from an ICH likely secondary to both amphetamine use and sexual intercourse. We have reviewed the guidelines and goals of care, particularly within the ED, established by the American College of Emergency Physicians (ACEP) and the American Heart Association (AHA) core concepts that must be well understood by an ED physician [1-2]. We have concluded with a brief discussion on amphetamine, its prevalence, and how it may lead to ICH. Given the known epidemic of amphetamine abuse with both increased access and low-cost production of highly potent crystalline methamphetamine in the world and the United States, this could be of high relevance to the ED physician.

## Case presentation

A 17-year-old male patient with no known medical history presented to a rural community ED with reports of seizure activity and altered mental status. The patient had sudden onset headache while having intercourse at home, which progressed to seizure activity witnessed initially by the patient’s girlfriend and then by his mother. These were described as tonic-clonic jerking, lasting only seconds. The patient has no known history of seizures. Of note, the patient does not currently take any medications nor does he have any allergies to medications. He has no known medical problems; however, both mother and daughter confirmed the patient's remote history of amphetamine use. On presentation, his vitals were as follows: heart rate, 102 beats per minute; blood pressure (BP), 145/83 mmHg; respiratory rate, 16 breaths per minute; and SpO_2_ of 98% on room air.

The patient presented to the ED via emergency medical services with an acute-onset seizure and altered mental status. An initial exam revealed an obtunded patient without response to verbal or painful stimuli but maintaining his airway with spontaneous respirations. Neurological exam was concerning for a left upward eye gaze with associated bilateral, non-fatiguing nystagmus. Initial differential diagnosis was broad with considerations of metabolic and electrolyte abnormalities, infection, and toxic ingestion; however, given the patient's abnormal neurological examination, intracranial pathology was the top initial concern. Blood was collected and sent to the laboratory with pertinent findings presented in Table 1.

**Table 1:** Laboratory test results WBC, white blood cell; INR, international normalized ratio

Analyte	Value	Reference Range
WBC (K/µL)	17.91	4.50–11.00
Hemoglobin (g/dL)	15.0	13.0–16.0
Hematocrit (%)	40.4	41–53%
Platelets (K/µL)	251	150–400
INR	1.1	1–1.5
Urine drug screen	Positive for amphetamines	Negative
Glucose (mg/dL)	179	65–99

**Figure 1:**
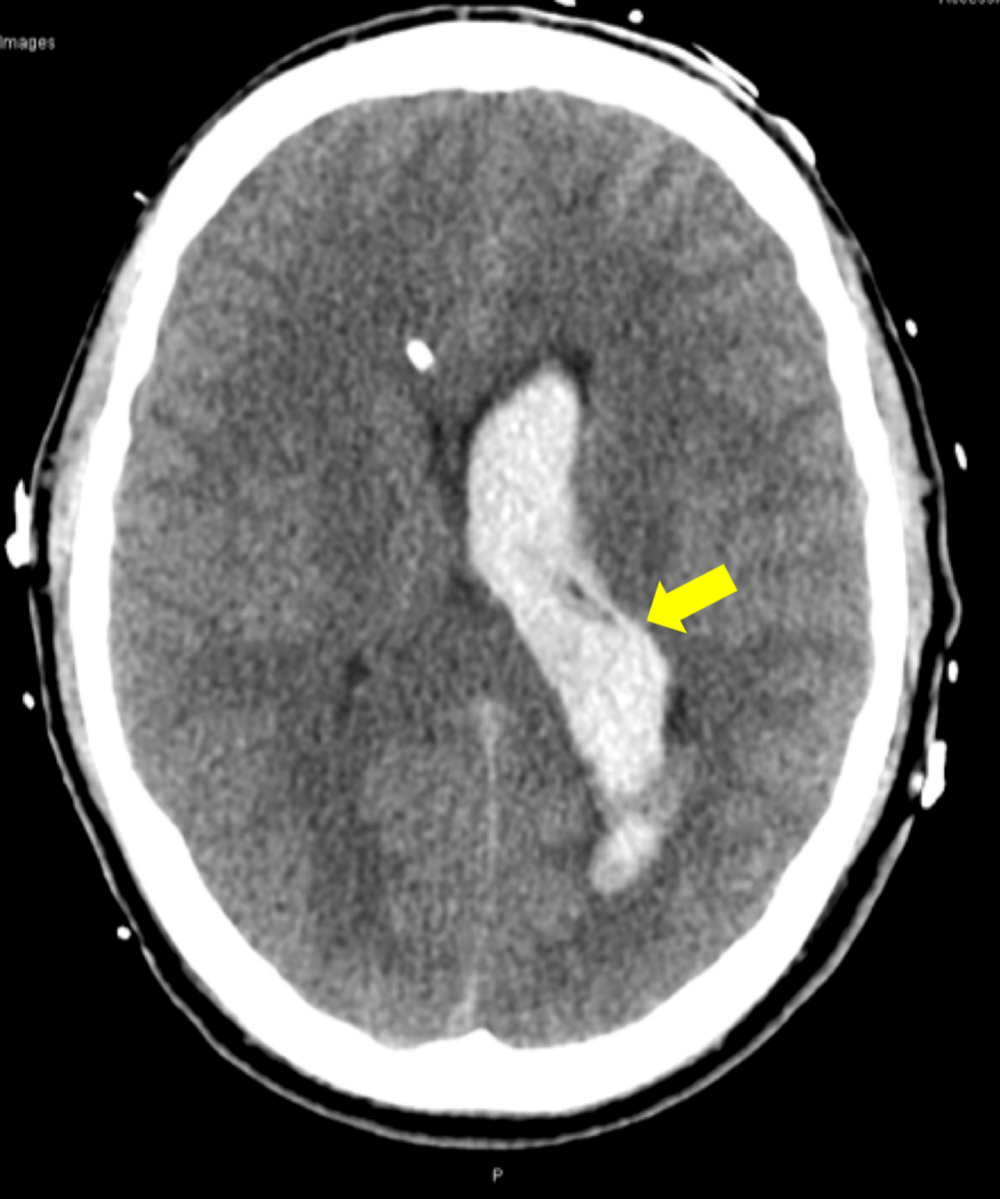
Transverse CT head image showing left intraventricular hemorrhage CT, computed tomography

**Figure 2:**
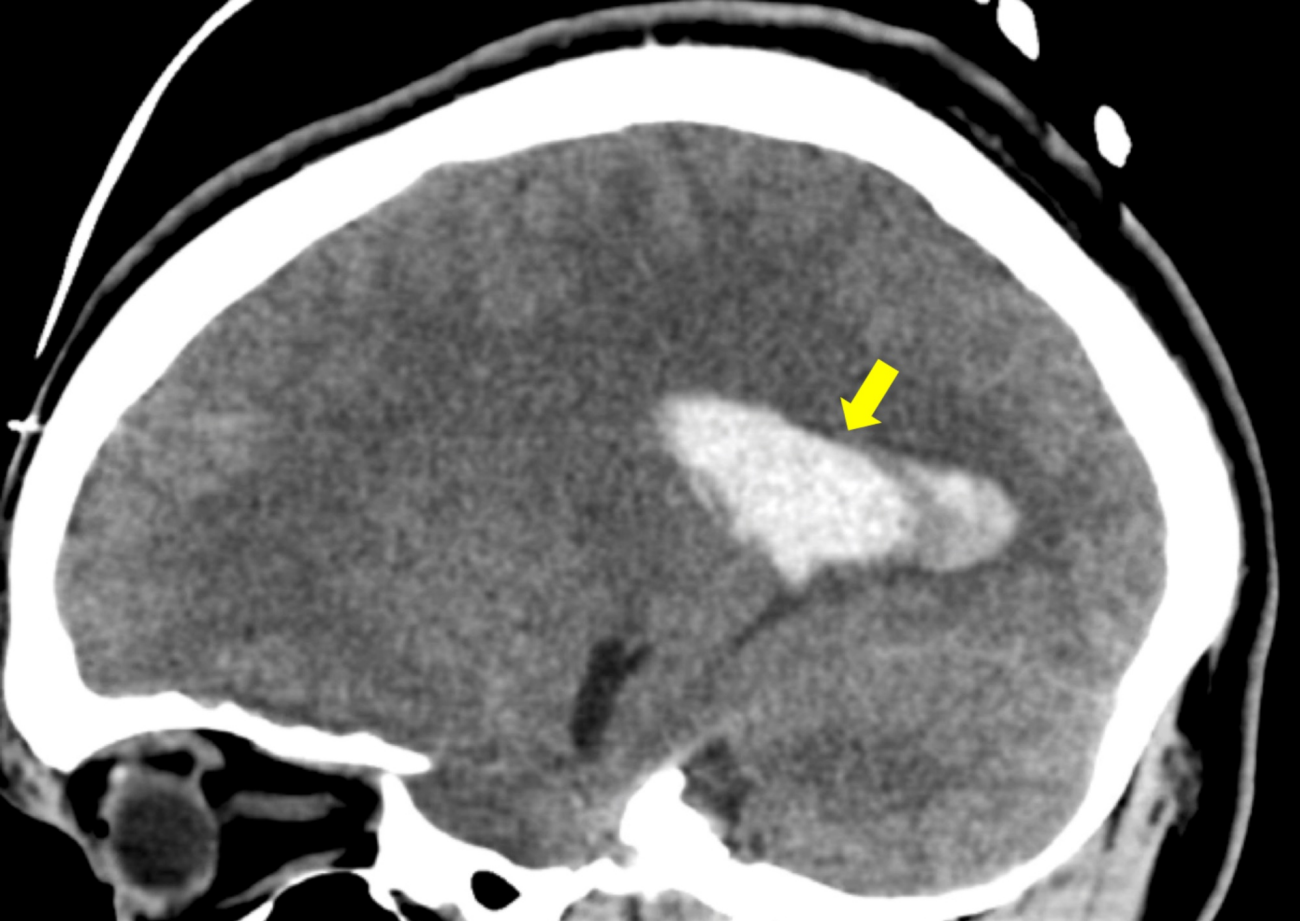
Sagittal CT head showing left intraventricular hemorrhage CT, computed tomography

Due to ongoing short episodes of seizure-like activity, the patient was administered nasal midazolam and was immediately sent for a non-contrasted head computed tomography (CT) scan (Figures 1-2). This showed a significant ICH with SAH with concern for developing hydrocephalus and possible temporal horn entrapment. Given the findings of ICH with SAH and concern for status epilepticus, the patient was intubated and started on a levetiracetam drip for seizure control and a propofol drip for both sedation and seizure control. Despite the propofol use, he remained hypertensive and was initiated on labetalol for targeted ICH BP management. Ultimately, this allowed for safe transfer to a tertiary care facility with neurosurgery capability.

Hospital course

Upon arrival at the tertiary facility, the patient remained intubated for airway protection. As noted above, the urine drug screen did return positive for amphetamines, which is postulated as the culprit for the spontaneous hemorrhage. During the patient’s stay, bilateral external ventricular drains (EVD) were placed to allow for simultaneous cerebrospinal fluid drainage and intrathecal tissue plasminogen activator administration. Extensive imaging and procedures were completed in hopes of identifying the etiology, including CT angiography and four-vessel angiography. Neither CT angiography nor invasive four-vessel angiography found an identifiable source of hemorrhage, as no aneurysms or vascular malformations were identified.

Clinically, the patient progressed from a comatose state on initial presentation to fully alert, oriented, and able to follow commands. On day eight of his hospital stay, he was successfully extubated. On day 14, which is known as the last day of the vasospasm window in the neurocritical care setting, the patient had the EVDs removed and was transferred to a neurocritical care step-down unit where he underwent physical therapy. He was discharged on day 28 without any focal neurological deficits. Follow-up non-contrast head CT revealed no further ICH.

## Discussion

SAH, by definition, is the extravasation of blood into the cerebrospinal fluid. It is identified with imaging, including a non-contrast head CT with further diagnostics, including a lumbar puncture, which goes beyond the scope of this discussion [5]. Incidence has remained the same in the past 30 years; however, mortality has decreased significantly with established guidelines, including early disposition to tertiary neurocritical care centers with neurosurgical and neuroendovascular capabilities and on-site neurointensivists [5]. A recent meta-analysis that used data from four studies and included over 360,000 patients showed a reduction in hospital mortality in patients treated in high-volume centers (odds ratio, 0.77; 95% CI, 0.60-.97). It is estimated that 85% of non-traumatic SAH are due to aneurysms; however, 15% are non-aneurysmal and are found to have an unremarkable angiogram. Although etiology is uncertain in the latter, the prognosis is much grimmer in the former. Early treatment measures aim to decrease the likelihood of neurological deterioration and secondary harm. Lord et al. found that early deterioration is often due to acute hematoma expansion, large volume edema, vasospasm, and delayed ischemia [1]. Later complications also include neuroendocrine disorders and impaired autoregulation leading to metabolic and electrolyte abnormalities.

Ultimately, these known complications drive the initial management within the ED. Dogma ingrained into training residents and well-established attendings hold true here. Airway, breathing, and circulation come first. In our case, our patient was intubated for airway protection. Further, the ACEP and AHA guidelines include cardiopulmonary monitoring and frequent neurological checks. The head of the bed must be propped to 30 degrees to allow for venous drainage. Blood pressure must be closely monitored. Theoretically, significantly elevated blood pressures will lead to acute rebleeding; however, pressures significantly lowered can lead to decreased perfusion pressure and ischemic events. In a retrospective study with goal pressures of less than 100 mmHg, systolic rebleeding was reduced to 15% vs. 33% in the nontreated group. However, the risk for ischemia doubled: 40% in the treated group vs. 22% in the non-treated group [6]. However, the 2012 AHA guidelines recommend a systolic blood pressure less than 160 mmHg, and early neurosurgical consultation and guidance are encouraged [7-8].

These findings bring us to the crux of this case report: an SAH in an otherwise young healthy male patient with the only known risk factor of remote polysubstance abuse and positive amphetamine result on the toxicological screen. A recent review of known hemorrhagic and ischemic events in the setting of illicit amphetamine abuse looked at 370 articles and included 87 hemorrhagic and 17 ischemic strokes. Roughly one-third of the hemorrhagic strokes resulted in lasting residual neurologic deficits, one-third with full recovery, and one-third resulting in death. Mechanisms of hemorrhage were postulated by the authors that the acute and chronic abuse of amphetamines leads to an acute elevation in blood pressure, vasculitis, direct vascular toxicity, vasospasm, and even necrotizing angiitis [2]. This phenomenon is highlighted as far back as 1975 in a case of a 19-year-old female patient who presented with a headache and neurological deficits and was found to have a ‘beaded’ appearance on arteriogram [4]. Subsequent imaging in our case revealed no definitive reason as to why a bleed occurred; however, combined intercourse and amphetamine use as the culprit cannot be ignored.

## Conclusions

Management of ICH secondary to an SAH in the ED does not acutely change whether the patient is a 17-year-old amphetamine user or a 72-year-old with a known saccular aneurysm. Early stabilization and mobilization to a tertiary care center is key to allow for definitive management. What must be understood and appreciated is that although our role as emergency physicians is to ensure early stability, we must still understand that this devastating occurrence can manifest and present in atypical ways.
